# Development and Pilot Evaluation of an Online Relapse-Prevention Program Based on Acceptance and Commitment Therapy for Chronic Pain Patients

**DOI:** 10.2196/humanfactors.3302

**Published:** 2015-01-05

**Authors:** Martine Fledderus, Karlein MG Schreurs, Ernst T Bohlmeijer, Miriam MR Vollenbroek-Hutten

**Affiliations:** ^1^Roessingh Research and DevelopmentEnschedeNetherlands; ^2^Faculty of Behavioural SciencesDepartment of Psychology, Health and TechnologyUniversity of TwenteEnschedeNetherlands

**Keywords:** chronic pain, eHealth, acceptance and commitment therapy, relapse prevention, aftercare

## Abstract

**Background:**

A significant number of chronic pain patients experience a decline in therapeutic effects after rehabilitation. As face-to-face contacts with health care professionals are not always feasible after treatment, new, innovative, fully automated relapse-prevention programs are highly needed.

**Objective:**

In this study an online, automated relapse-prevention program based on acceptance and commitment therapy (ACT)—both as a website and as a mobile app—was developed and evaluated. At each step of the development, end users (ie, chronic pain patients) were consulted in order to fully address their needs.

**Methods:**

In a step-by-step process, a contextual inquiry, requirement specification, and design were executed with chronic pain patients by conducting, respectively, a focus group (n=10), interviews with rapid prototyping (n=28), and a user- and expert-based usability evaluation (n=14). Furthermore, a pilot evaluation was conducted with 14 chronic pain or fatigue patients who had received the online relapse-prevention program following a multidisciplinary ACT treatment. They were interviewed about their usage and the usefulness of the program in supporting them to maintain changed behaviors and prevent relapses in avoidance and pain control behaviors.

**Results:**

The three stages provided information about the expected needs of end users, comments about the usefulness of the proposed features, and feedback about the design and usability of the program. This resulted in a fully operational, online relapse-prevention program. Results from the pilot evaluation showed that 9 patients used the online program after treatment, 5 of whom indicated that the program supported them after treatment. Of all the patients, 4 of them indicated that the program did not support them because they wanted more social interaction with other users.

**Conclusions:**

This study showed that an innovative, automated, online program that is user friendly can be developed by involving the end users in each step. The program was evaluated positively by some participants. The evaluation showed that the online relapse-prevention program has the potential to support chronic pain patients in maintaining their changed behaviors and preventing relapses in avoidance and pain control behaviors.

**Trial Registration:**

Nederlands Trial Register (NTR) Number: NTR4177; http://www.trialregister.nl/trialreg/admin/rctview.asp?TC=4177 (Archived by WebCite at http://www.webcitation.org/6Ur6EFD1D).

## Introduction

Multidisciplinary rehabilitation programs based on cognitive behavioral therapy (CBT) or acceptance and commitment therapy (ACT) for chronic pain patients have shown positive effects on the interference of pain in daily life and on physical and mental functioning [1-3]. However, a significant number of patients experience a decrease in the therapeutic effects one year after rehabilitation [4,5]. Providing support after treatment might help to generate the skills required to prevent or manage the occurrence of a relapse. However, face-to-face contacts with a health care professional are not always feasible in rehabilitation care due to limited therapist time and a lack of (financial) resources [6]. A relapse-prevention program based on eHealth might overcome these barriers because it offers the user more convenience and more control over the content and timing of the intervention [7]. Moreover, it might be more cost-effective than face-to-face treatment as guidance can be given through email or short message service (SMS) text messaging [8]. A growing number of studies have shown that Web-based CBT interventions are effective for the treatment of chronic pain [9,10]. Two studies concluded that Web-based, CBT relapse-prevention programs following multidisciplinary pain treatment have shown positive effects [6,11].

In this study we describe the development of a new, online relapse-prevention program—in the form of a website and/or mobile app—based on ACT. The main focus of ACT is enhancing psychological flexibility which includes the processes of acceptance and value-based behavior [12]. For chronic pain patients, acceptance means that one acknowledges the pain and abandons unproductive attempts to control the pain [13]. When these attempts are relinquished, an individual can choose, or persist in, behaviors that are in line with life values [14]. Values are important, chosen life directions, for example, in the domains of family, work, and social life. Clients are encouraged to perform actions which are in line with their values, regardless of what emotions or thoughts might occur. Other important processes of ACT are mindfulness and self-as-context. These processes help a person to consciously center themselves in the present moment. This grounded awareness in the present moment is a necessary premise to be open and flexible to experience, and to move toward valued, day-to-day life activities [14]. The content of the online relapse-prevention program was based on cognitive behavior models of relapse, mostly applied in the area of substance abuse [15]. The most critical predictor of relapse is the individual’s ability to perform effective coping strategies when facing high-risk situations. Therefore, relapses may be prevented by identification of these high-risk situations and teaching effective coping strategies. Other important determinants for preventing a relapse are high self-efficacy, functional social support, and positive affect [15].

However, despite the promising results of eHealth interventions for chronic pain, the effect sizes are often less than expected. One reason for this might lie in the low adherence rates of eHealth interventions, for instance, participants are not following the intervention as intended. For example, in the study of Moessner et al it was shown that a Web-based aftercare intervention following multidisciplinary therapy for chronic back pain had positive effects on pain intensity, but 70% of the patients did not adhere to the intervention [6]. Kristjánsdóttir et al only found a large effect size on the primary outcome (catastrophizing) in the participants who completed the mobile phone-based ACT intervention after a rehabilitation program for chronic widespread pain [16]. This corroborates earlier findings about the relationship between adherence and effect in psychological online interventions [17]. Studies on the underlying reasons for these low-adherence rates are scarce. However, commonly suggested reasons include shortcomings in the user-friendliness of the technology, problems with integrating the technology into day-to-day life, or a failure to tailor the intervention to the users’ real needs [18].

The first aim of this study is to develop an innovative, fully automated, online relapse-prevention program that enables rehabilitation centers to implement this program. For the development process, a user-centered design was used that might overcome the problems described above. A user-centered design has been shown to have positive effects, especially on user-satisfaction levels and on addressing user needs [19]. A user-centered design gathers feedback from potential users during the whole development and design process [20]. The development and design processes were guided by a roadmap for creating an eHealth technology that follows the principles of a user-centered design [21]. The roadmap provides an overview of the different steps that need to be addressed with an explicit focus on involving all relevant stakeholders at each step for ensuring that the technology is broadly supported. Based on a review of current eHealth frameworks, the roadmap dictates six principles regarding technology development: (1) it is a participatory process, (2) it involves continuous evaluation cycles, (3) it is intertwined with implementation, (4) it changes the organization of health care, (5) it should involve persuasive technology, and (6) it needs advanced methods to assess impacts. Based on these principles, the roadmap consists of five research and development activities, which are contextual inquiry, requirement specification, design, operationalization, and summative evaluation [21]. This roadmap has been successfully used to develop an online, ACT, Web-based intervention for depression [22] and chronic pain [23]. In the current study, we initially focused on the first three processes to develop a new, innovative, online relapse-prevention program for chronic pain patients. Addressing the last two processes of the roadmap, the app is implemented in daily practice (operationalization), and the degree of successful uptake and the impact on health-related outcomes are evaluated (summative evaluation). To gain insights into these processes, we performed a pilot study where patients underwent multidisciplinary treatment. Following treatment, we evaluated whether patients found the program helpful in maintaining behavioral changes and preventing relapses in avoidance and pain control behaviors.

## Methods

### Contextual Inquiry

In the contextual inquiry, intended users are asked for information about their needs for a technology. Contextual inquiry also examines how the technology may fit into the day-to-day life of the intended users [21]. In the current study, this information was obtained by a focus group discussion with chronic pain patients. A group of 10 female chronic pain patients who recently finished an 8-week, inpatient, multidisciplinary ACT program at a rehabilitation center in the Netherlands was invited to take part in the focus group discussion conducted by 2 researchers (KMGS, MF).

Respondents were asked to discuss what they believed would help them to prevent relapses and whether they would use a website and/or a mobile app as a relapse-prevention program. They were also asked whether they wanted to have guidance by email or SMS text message. This focus group session was audiotaped with the permission of the respondents. The audiotapes were transcribed and were analyzed qualitatively by the researcher by summarizing common themes.

### Requirement Specification

**Figure 1:**
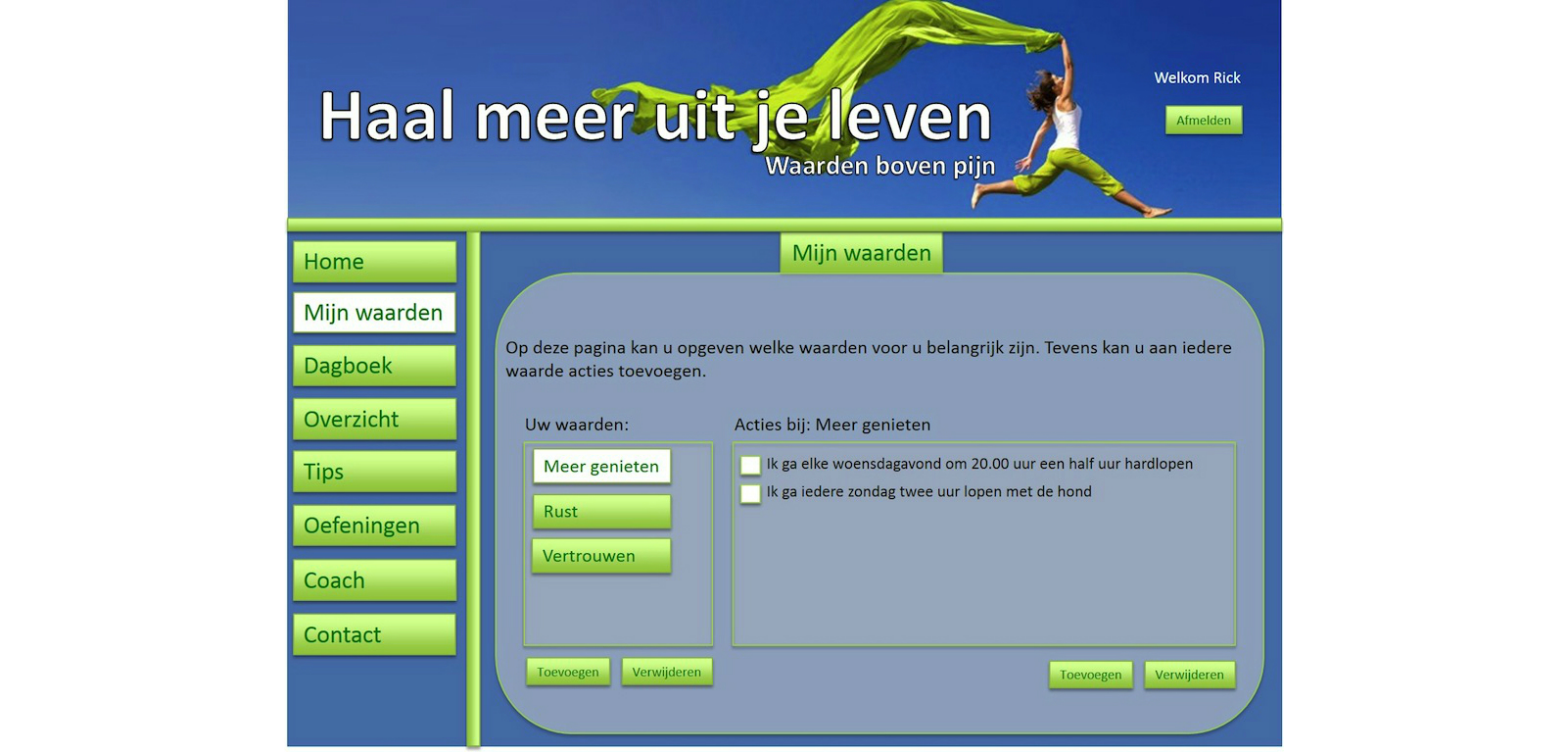
Example of a website prototype (Values and actions).

**Figure 2:**
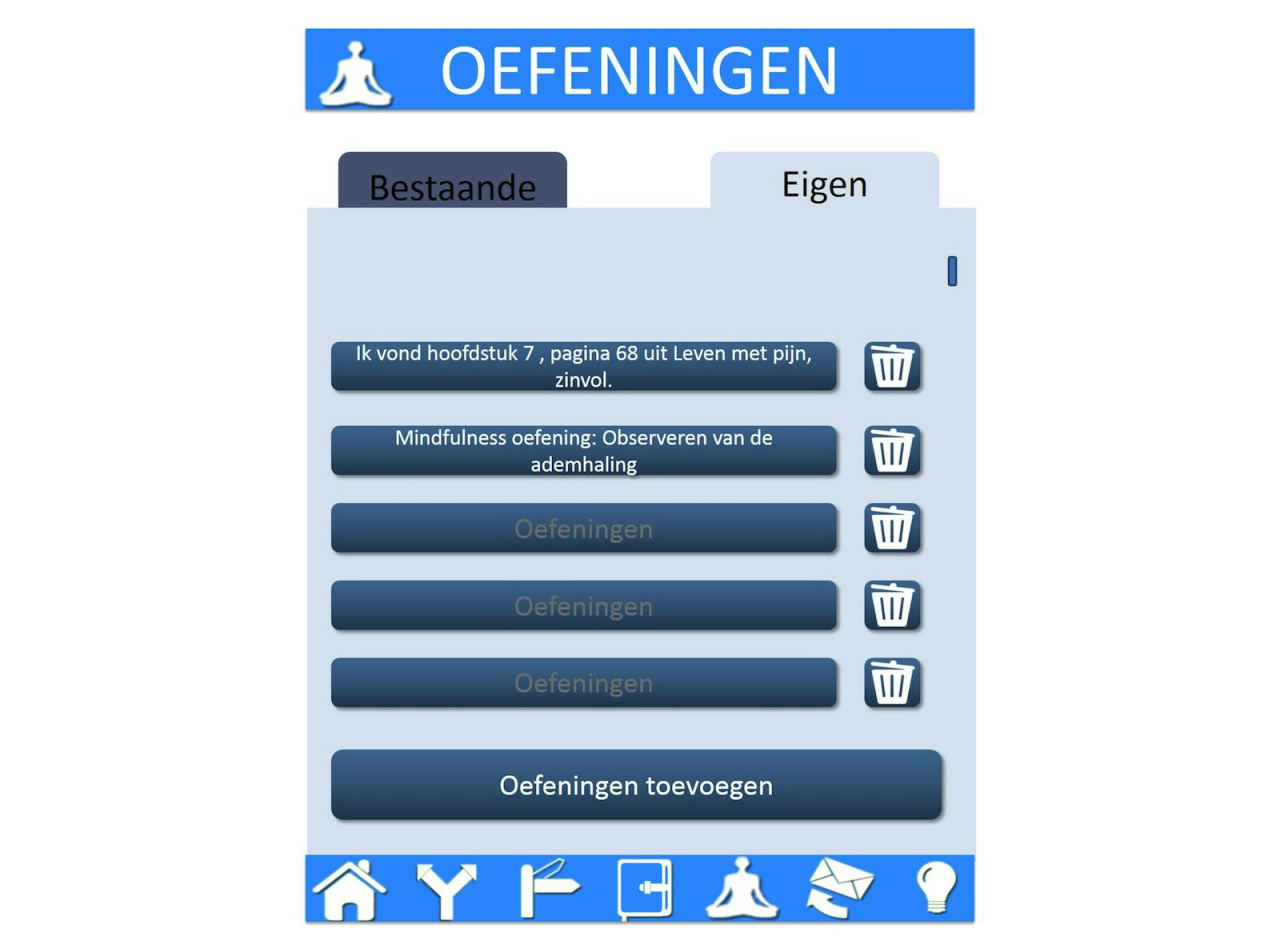
Example of a mobile app prototype (Exercises).

In the requirement specification activity the expected needs are translated into requirements of the technology [21]. Therefore, based on the expected needs that were identified in the contextual inquiry, a prototype of a website page and various prototypes of pages of a mobile app were designed using PowerPoint. On multiple slides, five features of the online program were presented to the participants. For examples of these prototypes, see Figures 1 and 2. There were five features that demonstrated what a potential user could do in the online relapse-prevention program:

1. Values and actions: Users could add or change various life values and corresponding actions.

2. Diary: The diary is for monitoring value-based living and included one question that asked whether the user had lived according to his/her values on a rating scale from 1 to 10. The answers to this question were presented in a chart including positive smileys if a score of 6 or more was achieved.

3. Exercises: Users could add various ACT exercises or search in a database that contains all the exercises included in the treatment.

4. Tips: Users could add their own tips, share these tips with other users, or see shared tips from other users.

5. Coach: Various options of guidance with SMS text message or email were shown to the participants and they could all choose which text message they would like to receive: (1) a reminder SMS text message to use the diary or to use the program itself, (2) a motivational SMS text message written by a health care professional, (3) a self-composed motivational SMS text message which could be sent at a later date, or (4) a motivational SMS text message tailored to particular scores that are obtained from the diary—this tailors the content more to the individual situation.

To obtain the requirements, semistructured interviews combined with the prototypes of the eHealth technology were conducted with chronic pain patients [24]. Therefore, 28 chronic pain patients that recently completed the multidisciplinary ACT program at the rehabilitation center were interviewed by 3 psychology students from the University of Twente. Mean age was 43 years (SD 11) and most were female (25/28, 89%). The interview scheme was focused on the usefulness of the five different features of the online program by showing the prototypes based on these features. We asked the participants to comment on the design of these features by asking them to describe their first impression in three words and to indicate whether they found what was shown to them to be clear. If participants found the feature useful, they were asked to indicate the exact moment—during and/or after treatment—at which they would use these features. Finally, questions were asked about how they would like to receive the guidance (Coach). The audiotapes were transcribed and analyzed by the researcher using an inductive thematic analysis [25]. For this analysis the data were reviewed for identifying relevant patterns (themes) in the data. Initial codes were given to the responses of the participants and these codes were collated to potential themes. After this step the themes were again reviewed by checking the whole dataset and refined if necessary.

### Design of the Technology

**Figure 3:**
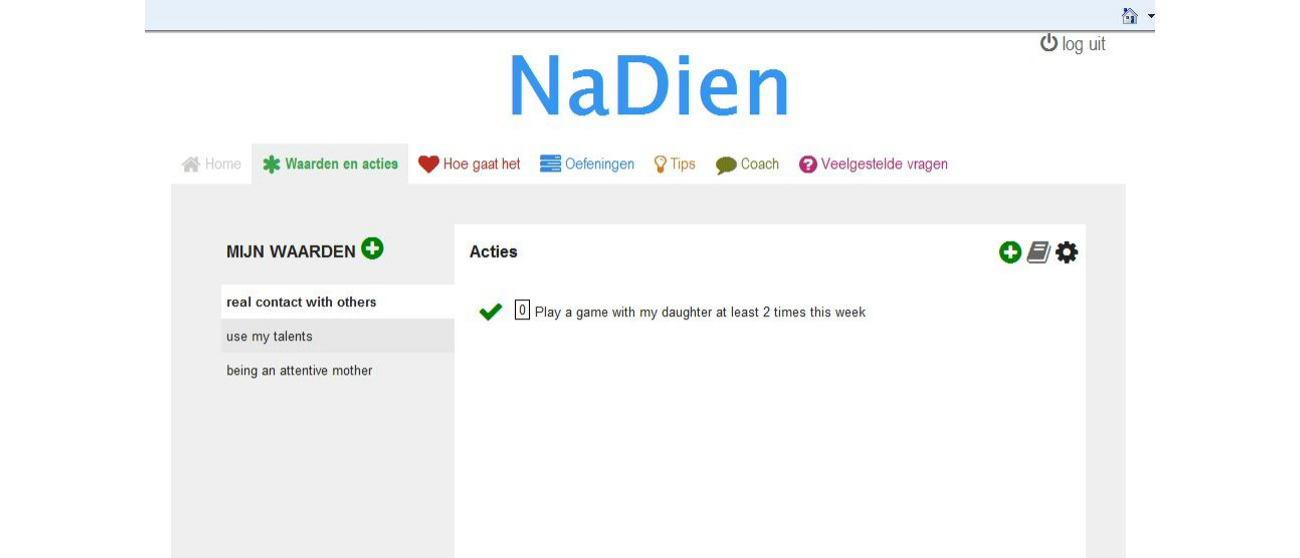
Screenshot of a page of the website (Values and actions).

**Figure 4:**
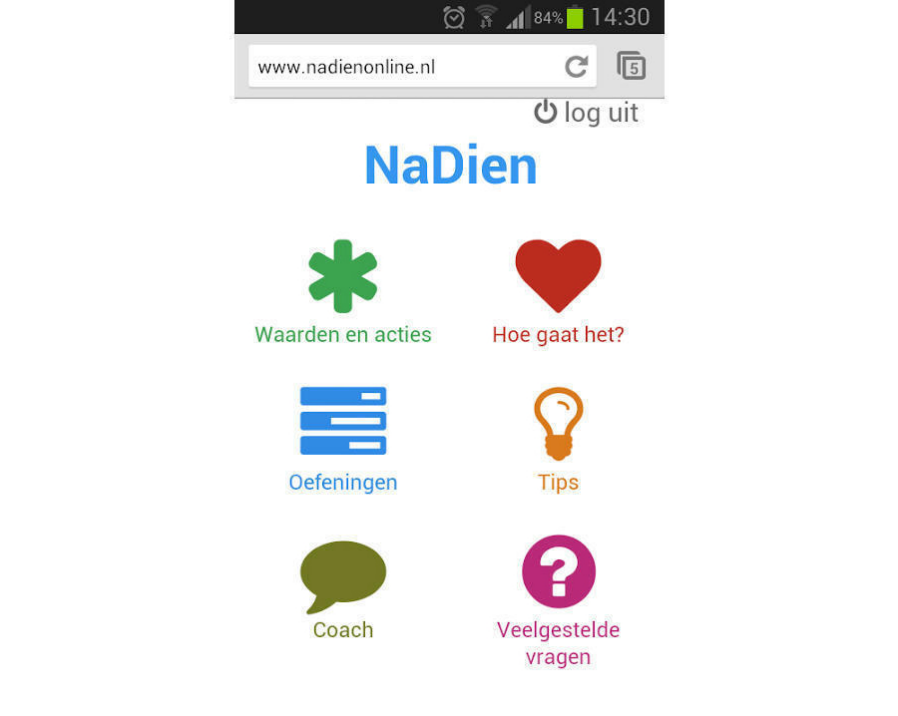
Screenshot of the mobile app homepage.

A fully operational program, both as a website and as a mobile app, was developed. For examples of these, see Figures 3 and 4. Based on the requirement specification, some changes were made. A library with examples of actions was added to the feature Values and actions. The name “Diary” was changed to “How are you?” and some extra tips written by a health care professional were added. For the Coach feature, several options for sending texts—both as email and SMS text message—were developed and users were free to choose between these options or to change them during use. Users can receive reminder messages after one week of not logging in or filling out the question in the “How are you?” section. The program includes an agenda for setting a fixed date and time to send the message. Users can receive a motivational message either once a week at random or within 24 hours after answering the question in the “How are you?” section. Furthermore, there was an option for the user to send self-composed motivational messages which could be sent once a week at random after treatment. All the reminder and motivational messages were written by 2 health care professionals (MF, KMGS). KMGS is a health care psychologist and is registered as a cognitive behavioral therapist with ample experience in ACT. She is a therapist in the multidisciplinary ACT team. MF has a PhD in psychology. Based on earlier knowledge, both in practice and earlier studies [23], they wrote all the messages in advance. The messages were programmed to be sent at random in the online program.

In this phase, the quality of the design was examined by evaluating its usability. Consequently, a user-based and expert-based evaluation method using a scenario-based think-aloud protocol was performed [24]. Usability tests with 5 chronic pain patients and 9 experts were conducted by 3 psychology students from the University of Twente. There were 5 target-group experts, such as a physiotherapist and psychologist who were working with chronic pain patients. Furthermore, there were 4 usability experts who were conducting research in eHealth. The mean age of the participants was 38 years (SD 12) and most of them were female (12/14, 86%). The participants were guided through the program using scenarios that included several tasks or problems that had to be solved by the user, such as “You want to add a new personal value to the program: how would you do that?” Participants were asked to verbalize their thoughts while they were taking part in these scenarios. The audiotapes were transcribed and the comments were defined as problems encountered and suggestions for improvements. A coding scheme based on system quality, content quality, and service quality was used [21,26]. System quality is defined as the user-friendliness of the program and the presentation of the content, such as the layout and where the buttons are placed. Content quality refers to the meaningfulness of the information. In other words, are the texts easy to understand and complete? Service quality refers to the degree to which the user assesses the service as being adequately provided, such as the perceived usefulness of the features and the provision of feedback [21,26]. Out of 14 participants, 8 of them (57%) evaluated the website and 6 of them (43%)—1 patient, 1 target-group expert, and 4 eHealth experts—evaluated the mobile app. Every participant could choose whether he/she wanted to evaluate the website or the mobile app. More eHealth experts evaluated the mobile app than patients. This might be explained by the fact that eHealth experts were more familiar with (mental health) apps at that moment. Every comment was evaluated as positive, negative, or neutral.

### Pilot Evaluation of the Online Relapse-Prevention Program

Based on the results of the usability evaluation, the system quality was improved and a manual was developed to give the users more information, for example, whom the tips were shared with. A pilot user evaluation was conducted with patients who received the online program after a multidisciplinary pain treatment for 2 months. This study was approved by an independent Dutch medical ethics committee (Medical Research Ethics Committee Twente, no. P13-07) and recorded in the Dutch primary trial registry for clinical trials (Nederlands Trial Register, NTR4177).

Inclusion criteria were chronic pain or fatigue patients who were following an inpatient 8- to 12-week, group pain treatment which started in March or April 2013 at the Pain Department of the Roessingh Rehabilitation Centre. Chronic pain and chronic fatigue patients receive the same multidisciplinary treatment in the rehabilitation center. Patients needed to have access to the Internet through a computer and/or a mobile phone at home. The information letter described that it was recommended to use the online program for 5 to 10 minutes daily. The researcher had access to the actual accessed data use of the program, including how many times the participants were logged in and which features they had used, so self-report data use could be compared to the actual usage. In the fourth week of their treatment, the researcher explained the study and the patients received a letter with information about the study and a letter requesting their informed consent. In the sixth week of the treatment, patients who agreed to participate handed over the signed informed consent, were given access to the online ACT relapse-prevention program, and received a short introduction to the program. Following a period of 2 months after the group treatment, all of the participants were invited for a telephonic, structured interview conducted by the researcher (MF). The interviews were audiotaped with the permission of the participants and took, on average, 20 minutes to complete. The interview scheme started with a general question on the helpfulness of the online ACT relapse-prevention program in supporting them with the actions they intended to carry out after treatment. Participants were asked how many times they had used the program and whether they intended to continue using the program. The interview continued with an open discussion to identify which parts of the intervention they found to be most useful. Furthermore, they were also asked whether they would recommend this program to other patients. The interview ended with the question “Do you have any recommendations for improving the program?”

In total, there were 5 groups that started with the pain treatment with a total of 27 patients. All of them were given a letter with information about the program and a form requesting their informed consent. The informed consent form was signed by 17 patients and they subsequently received the online program. The mean age of the participants was 38 years and most of them were female (14/17, 82%). All 17 patients were invited for the interview and 14 of them were interviewed by the researcher. The reasons for not taking part in the interview were medical (1/17, 6%), feeling very good (1/17, 6%), and unknown (1/17, 6%).

## Results

### Contextual Inquiry

All participants indicated that there is a need for an aftercare program and they would all like to have contact with their health professionals after treatment, for instance, through a telephone call or individual face-to-face contact. Most respondents would use an eHealth program, such as a website or mobile app. In this online program 8 participants out of 10 (80%) found it useful to register their values and actions and to read ACT-based exercises. Of the 10 participants, 4 of them (40%) thought it would be useful to make a start with completing these exercises and registering their values and actions during treatment. All participants would like to have contact with other patients to share tips and to give each other pep talks. Of the 10 participants, 2 of them (20%) proposed a forum for facilitating this contact with other patients. All participants would like to receive reminders by SMS text messaging or email from a health care professional to help them adhere to value-based behavior after treatment. Of the 10 participants, 8 of them (80%) indicated that reminders could be preformulated and sent automatically, preferably at a higher frequency rate and directly after the end of the treatment. Of the 10 participants, 3 of them (30%) also thought that it would be helpful to add their own motivational texts during treatment in the online program that can be sent after treatment. Additionally, 2 participants out of 10 (20%) indicated that the text messages should be tailored to the individual patient’s personal situation.

### Requirement Specification

Almost all of the participants indicated that they would use the online aftercare program (27/28, 96%). Some stated that they would like to use a website (12/28, 43%), a combination of a website and a mobile app (9/28, 32%), or only a mobile app (7/28, 25%). In general, participants found the design of the five features to be clear and convenient. Table 1 shows an outline of the participants’ evaluation of the usefulness, the expected use, and needs of the various features of the online program. Almost all of the participants assessed all the features as useful and they indicated that they would use these features mostly during and after treatment. Furthermore, as can be seen in Table 1, the expected needs concerning the Coach feature are further specified and some improvements were given, such as changing the term “Diary”.

**Table 1:** User evaluation of the features of the online relapse-prevention program (n=28).

Features	Usefulness, n (%)	Use, n (%)	Moment of use	Expected needs
Values and actions	28 (100%)	26 (93%)	During and after treatment.	Database with some examples of actions.
Diary	28 (100%)	25 (89%)	After treatment.	Changing the name “Diary”.
Exercises	28 (100%)	27 (96%)	During and after treatment.	Database with some examples of exercises.
Tips	28 (100%)	27 (96%)	During and after treatment.	Tips from a health care professional.
Coach	28 (100%)	28 (100%)	After treatment.	Preference for receiving an SMS text message instead of an email. Receiving a reminder for the diary and technology. Choosing the frequency and moment to send out the reminders. Receiving an SMS text message with motivational content written by the health professional at random. Receiving an SMS text message with motivational content written by health professional after filling out the diary. Receiving a self-composed SMS text message.

### Design of the Technology

All comments are represented in Table 2. They were largely the same between users and experts. The most positive comments were about the quality of the system. In particular, the ease of use of the website/app (eg, clear navigation, clear buttons, simple) and the design (eg, fresh, calm) were rated positively. The other most frequently mentioned positive comments were about the quality of the service, namely the perceived usefulness of the features (eg, adding personal values, sharing tips with other users, the options of receiving an SMS text message). The most negative comments were about the quality of the system as some technical errors occurred or icons were unclear. For example, the icon for sharing the tips was unclear because the participants did not recognize this icon. In the Diary feature, it was unclear that to register you had to slide the bar instead of clicking. The negative comments about the quality of the content were mostly linked to the Tips feature since the participants did not know with whom the tips were shared and, therefore, they did not want to use this feature.

**Table 2:** Number and positivity or negativity of comments yielded from the user- and expert-based methods.

Property of program	Number of comments by users^a^			Number of comments by experts^b^			Total
	+^c^	+/-^d^	-^e^	+	+/-	-	
System	45	5	22	52	10	65	199
Content	2	3	8	0	4	19	36
Service	15	0	3	11	2	10	41

^a^There were 5 users.

^b^There were 9 experts.

^c^Positive comment.

^d^Neutral comment.

^e^Negative comment.

### Pilot Evaluation of the Online Relapse-Prevention Program

#### Overview

Table 3 shows the results of the pilot evaluation. There were 9 participants out of 14 (64%) who used the program and all of them will continue using the program. Out of 14, 5 participants (36%) never used the program at all, but most of them (3/5, 60%) indicated that they were going to use the program in the near future. Self-report data on the usage of the program were comparable to the actual accessed data usage. Almost all of the participants indicated that they would recommend the program to other patients (12/14, 86%). Furthermore, most participants (10/14, 71%) indicated that it would have been useful if they could have already started using the program during treatment, particularly with regard to completing certain parts of the program (eg, Values and actions, Tips), discussing it with other patients, and becoming better acquainted with it.

**Table 3:** Results of the pilot evaluation.

Participants (n=14)			n (%)
**Nonusers**			5 (36)
	Preference for use during treatment		4 (29)
	Recommend to other patients		5 (36)
	Use in future		3 (21)
	**Most useful feature**		
		Motivational messages	2 (14)
**Users with positive evaluation** ^a^			5 (36)
	Preference for use during treatment		3 (21)
	Recommend to other patients		5 (36)
	Use in future		5 (36)
	**Most useful feature**		
		(Mindfulness) exercises	4 (29)
		Motivational messages	3 (21)
		Tips	3 (21)
**Users with negative evaluation** ^b^			4 (29)
	Preference for use during treatment		3 (21)
	Recommend to other patients		2 (14)
	Use in future		4 (29)
	**Most useful feature**		
		Motivational messages	3 (21)
		Tips	1 (7)

^a^The online program was supportive in maintaining behavioral changes and preventing relapses in avoidance and pain control behaviors.

^b^The online program was not supportive in maintaining behavioral changes and preventing relapses in avoidance and pain control behaviors.

#### Nonusers

Reasons for nonuse were feeling well at that particular moment (3/14, 21%), a medical reason (1/14, 7%), and having other priorities (1/14, 7%). Of the 5 nonusers, 2 of them (40%) received motivational messages and found them very pleasant and supporting. Of the 5 nonusers, 3 of them (60%) thought they would use the program in the near future, especially in the case of a relapse (2/5, 40%). No suggestions for improvements were given.

#### Users With a Positive Evaluation

Out of the 14 participants, 5 of them (36%) indicated that the online program supported them in their efforts to perform their intended actions. Out of these 5 participants, 2 of them (40%) used the online program regularly (eg, at least once a week) and the other 3 participants (60%) used it 3 to 5 times in total. The online program functioned as a summary of the treatment and reminded them of what they had learned during treatment. The (mindfulness) exercises, the motivational messages, and the tips were evaluated as being “useful” and “very pleasant” as they functioned as reminders about the treatment. One participant recommended changing the question “How are you?” to more personal actions.

#### Users With a Negative Evaluation

Of the 14 participants, 4 of them (29%) indicated that the program did not support them after treatment. Of these 4 participants, 3 of them (75%) used the program 1 to 3 times, and 1 of them (25%) used the program at least once a week. All 4 participants out of 14 (29%) indicated that they missed the interaction with other patients in the program, for example, using a forum to share experiences with other users. Participants were satisfied with the motivational messages and the exercises because they served as a reminder for the treatment. They will continue to use the program, particularly the motivational messages (3/4, 75%), as well as the tips and the values and actions (1/4, 25%).

## Discussion

### Principal Findings

In this study, a new, innovative, online relapse-prevention program for chronic pain patients based on acceptance and commitment therapy was developed and evaluated. The first aim of the study was to develop an automated, user-friendly, online relapse-prevention program that fulfills the needs of chronic pain patients. A contextual inquiry by focus group discussion, requirement specification by interviews with rapid prototyping, and a user- and expert-based usability evaluation of the fully operational program successively provided the input for the next step in the development process. The chronic pain patients reviewed the program from their points of view and context, both on design and content for ensuring that the eHealth program was usable and acceptable. Accordingly, our user-centered developmental process resulted in a program with a simple design, large icons, and few layers of information and text. A pilot evaluation with chronic pain or fatigued patients who had received the online relapse-prevention program following a multidisciplinary ACT treatment (n=14) showed that this development process was satisfactory. Two-thirds of the participants (9/14, 64%) used the program in the 2 months after treatment. They did not have new suggestions on the usability of the program and nonusers (5/14, 36%) stated that their nonuse was not caused by complexity or inadequate usability of the program. This probably improves the uptake of the online relapse-prevention program, as many problems with adherence to eHealth programs are due to complexity and inadequate usability [18].

Furthermore, the user-centered development process resulted in information on the needs concerning the content of the program. The resulting program consists of the essential building blocks of a relapse-prevention program [15]. Firstly, participants have to recognize situations with a high risk of relapse. In ACT, relapse is defined as falling back on pain-avoidance behaviors instead of performing values-based actions [13]. By registering their values and actions and by monitoring values-based behavior with the tool “How are you?” participants are reminded of their important values and actions and recognize when they are relapsing to pain-avoidance behaviors. Next, patients have to have adequate coping skills to face high-risk situations. In the program, participants have access to descriptions of all exercises from which they learned these coping skills, their own favorite exercises, and tips from fellow users. Finally, social support after treatment was evaluated as an important component for the online relapse-prevention program. Social support can provide relevant shared advice and facilitates the process of finding recognition [27]. The program provides social support by offering the opportunity of sharing tips and by sending motivational text messages. Earlier research showed that text messages can be very effective for providing reminders or feedback for achieving a behavioral change [28].

The second aim of the study was to evaluate whether the online program supported patients after treatment in maintaining their changed behaviors and preventing relapses in avoidance and pain-control behaviors. The results of the pilot evaluation showed that, most of all, the motivational messages and the exercises were evaluated as very useful and pleasant. Results from the pilot evaluation showed that 9 patients out of 14 (64%) used the online program after treatment, 5 (5/9, 56%) of whom indicated that the program supported them after treatment. In general, the adherence rates and the evaluation of the program were disappointing. Although it was recommended to use the program daily, the 9 participants out of 14 (64%) that actually used the program only used it once a week or 1 to 5 times in total. Furthermore, 5 out of 14 (36%) participants never used the program. Of these 5 nonusers, 3 (60%) of them indicated that they will use the program in the near future, but we do not know whether these participants really intended to use the program or whether they were providing socially desirable answers. Our results corroborate with earlier results on low adherence rates in online programs for chronic pain patients [6,16]. Daily use of the online relapse-prevention program is essential to assess high-risk situations and to monitor the degree of values-based behavior. Earlier research has shown that there is a relationship between adherence and effect in psychological online interventions [17]. Also in this study, the participants with positive evaluations did use the program more often than the participants with negative evaluations.

A possible explanation for the low adherence in this study is that most patients had an initial preference with using the program during treatment, particularly with regard to registration of values and values-based actions. For further use, we recommend integrating the program into the multidisciplinary pain treatment program and to train health care professionals to inform and motivate patients to use the program during treatment. Furthermore, the program is not yet successful in providing sufficient social support. The 4 participants out of 14 (29%) who stated that the program was not helpful in the period after treatment indicated that they missed interacting with other participants. Therefore, it is advisable to implement a function where users can react to the shared tips.

### Limitations

Limitations of this study included the small samples of users involved in each step and the recruitment of all participants from the same rehabilitation center. Although generalization has to be made with care, the number of participants concurs with the recommended numbers by other studies [29,30]. Another limitation is that we did not involve all stakeholders in each step, such as patients, care providers, managers, and information and communications technology (ICT) developers [21]. However, we worked together closely with the rehabilitation center by informing all the relevant care providers about the results of the steps and by involving the health care professionals in the development process. Finally, in the evaluation of the online program, we did not examine the number of participants that experienced residual problems before and after the relapse-prevention program. For a more effective trial of the online relapse-prevention program, it is important to monitor the effects of the program on relapses.

### Future Work and Conclusions

Relapse prevention is still a topic that is hardly examined in the area of chronic pain [4]. This study strengthens the call for further attention for this topic as almost all participants in each step of the development process indicated that they would use a relapse-prevention program after their treatment. Although we provided some insight into the processes of operationalization and summative evaluation of the roadmap, it is relevant to improve a number of conditions in the rehabilitation center for full implementation, such as training health care professionals and integrating the online program into the treatment. An important next step is to evaluate the online relapse-prevention program based on its effectiveness at maintaining the positive effects after multidisciplinary treatment and preventing relapses. Besides measuring relevant health-related measures, it is important to further examine the usage. This will provide information on how to redesign or refine the eHealth technology for achieving higher effects. To conclude, this study provided an overview of how one can design a new, innovative, online relapse-prevention program and revealed valuable insights into the adaptations that have to be made to successfully implement the program in health care.

## Abbreviations

ACT: acceptance and commitment therapy
CBT: cognitive behavioral therapy
ICT: information and communications technology
SMS: short message service
